# Chronic myelomonocytic leukemia as a cause of fatal uncontrolled inflammation in familial Mediterranean fever

**DOI:** 10.1186/s13023-015-0295-9

**Published:** 2015-06-16

**Authors:** Fawaz Awad, Sophie Georgin-Lavialle, Anne Brignier, Coralie Derrieux, Achille Aouba, Katia Stankovic-Stojanovic, Gilles Grateau, Serge Amselem, Olivier Hermine, Sonia-Athina Karabina

**Affiliations:** Sorbonne Universités, UPMC University Paris 06, INSERM UMR_S933, Hôpital Armand-Trousseau, Paris, F-75012 France; Centre de référence de la fièvre méditerranéenne familiale, DHU I2B, Service de médecine interne, Hôpital Tenon, Assistance Publique-Hôpitaux de Paris (AP-HP), Paris, France; Service d’Hématologie clinique, AP-HP, Hôpital Necker, Université Paris Descartes – Sorbonne Paris Cité, Imagine Institute, INSERM UMR 1163 et CNRS ERL 8254, Paris, France; Laboratoire d’hématologie biologique, Hôpital Necker, 149 rue de Sèvres, 75015 Paris, France; Service de Génétique, Assistance Publique-Hôpitaux de Paris (AP-HP), Hôpital Trousseau, F-75012 Paris, France

**Keywords:** FMF, *MEFV*, Inflammation, CMML, Interleukin inhibitors, Monocytes, Interleukin 18

## Abstract

We report on a familial Mediterranean fever (FMF) patient homozygous for p.M694V in the *MEFV* gene who developed chronic myelomonocytic leukemia (CMML) leading to an uncontrolled and fatal inflammatory syndrome. Plasma levels of IL-6 and IL-18 were found to be very high, as compared to healthy controls and CMML-free FMF patients.

Our study unveils the interplay between two different disorders involving the same target cells, suggesting that in myelodysplasia with inflammatory manifestations, mutations in genes causing autoinflammatory syndromes, like *MEFV*, can be present and thus could be sought. Early chemotherapy with interleukin inhibitors could be proposed in such unusual situations.

**Electronic supplementary material:**

The online version of this article (doi:10.1186/s13023-015-0295-9) contains supplementary material, which is available to authorized users.

## Correspondence

Letters to the Editor:

Familial Mediterranean fever (FMF) is an autosomal recessive autoinflammatory disorder caused by mutations in the *MEFV* gene, mainly p.M694V in exon 10 [1, 2]. It is the commonest hereditary fever syndrome with recurrent episodes of fever accompanied by abdominal, chest and joint pain. *MEFV* encodes pyrin, a protein expressed in neutrophils and monocytes [3] and is involved in the regulation of inflammation. Daily and life-long colchicine administration can prevent both attacks and occurrence of inflammatory amyloidosis. *Ex-vivo* studies performed with monocytes from FMF patients have demonstrated the importance of increased secretion of the potent pyrogenic cytokine interleukin (IL)-1β. Subsequently, IL-1 inhibitors have been proposed as alternative or supplementary treatment in colchicine-resistant patients or in those presenting adverse events [4]. In addition, blocking the IL-1 pathway is safer, in terms of infectious risk, in comparison with other biological treatments [5, 6].

Chronic myelomonocytic leukemia (CMML) is a clonal hematopoietic stem cell disorder classified as a myelodysplastic/myeloproliferative neoplasm [7, 8]. CMML is characterized by absolute monocytosis (>1 × 10^9^/L) in peripheral blood persisting for at least 3 months [8]. The median age of CMML diagnosis is 70 years [7] and current treatment includes hydroxyurea and/or 5-azacitidine [8].

**Fig. 1:**
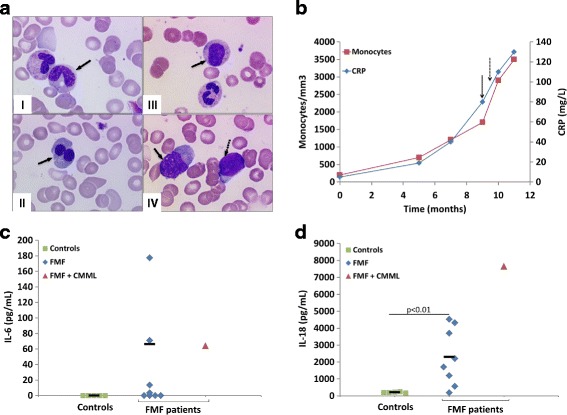
Morphological features of CMML in patient #9 and cytokine profiles in patients and controls. **a** Bone marrow smears from patient #9 with CMML and FMF (see Additional file 1: Table S1) (I) showing dysgranulopoiesis (hypogranular cytoplasm and Döhle bodies indicated with the arrow) at t = 0; (II) dysplastic binucleate erythroblasts at the stage of myelodysplastic syndrome (t = 0); (III) persistence of myeloid dysplasia (indicated with an arrow) at t = 6; (IV) appearance of a monocytosis (solid arrow) with excess of blasts (dashed arrow) when progression to CMML at t = 6. **b** Blood monocyte counts and serum CRP levels at the indicated time points in patient #9. t = 0 is first time the patient complained about symptoms worsening. The solid black arrow indicates the time of CMML diagnosis and the dashed arrow indicates when plasma was drawn for cytokine measurements. **c** IL-6 levels in plasma samples from patient #9, FMF patients and apparently healthy controls, as quantified by ELISA. **d** IL-18 levels in plasma samples from patient #9, FMF patients and apparently healthy controls, as quantified by ELISA

We report a case of an 84-year-old man who had typical FMF since his childhood. The diagnosis of FMF was confirmed by identification in the *MEFV* gene of the M694V mutation in the homozygous state. Lifelong colchicine therapy (1 mg/day) for 40 years abolished febrile crises. No other medical problem was reported. At the age of 83 (t = 0), he started complaining of general weakness and a blood test showed profound macrocytic anemia, with no other cytopenia (Hb = 7.2 g/dL; MCV = 104 fL; leukocytes 7.6 × 10^9^/L; platelets 228 × 10^9^/L). Additional laboratory tests showed no signs of hemolysis, inflammatory disease, hypothyroidism or deficiency in vitamin B12 or B9. Thus, a bone marrow smear was performed and revealed refractory anemia without blast excess (Fig. 1a, I&II) and with a normal karyotype. He received iterative red blood cell transfusions and vitamin D.

Six months later (t = 6), he presented with asthenia, fever and biological inflammatory syndrome although he took the same dose of colchicine. Serum amyloid A (SAA) as well as C-reactive protein (CRP) levels were elevated (226 and 20 mg/L respectively), and Hb was low (6.9 g/dL). Neither infection nor profound neoplasia was detected. He had no proteinuria, and salivary gland biopsy revealed no amyloidosis. Colchicine was increased to 1.5 mg per day, but because of occurrence of diarrhea –a well-known side effect of colchicine therapy–, the dose was finally maintained 1mg/day. During the following 3 months, his general status got worse: he lost 10 kg, fever and inflammatory syndrome persisted and required weekly red blood cell transfusions because of severe anemia (Hb = 5.5 g/dL). His condition suddenly deteriorated, with elevated fever and fatigue. Further investigations showed a progressive increase in monocyte count above 1 × 10^9^/L with persistent anemia (Hb = 6.8 g/dL) and severe inflammatory syndrome (SAA = 327 mg/L; CRP 80 mg/L; Fig. 1b). A new bone marrow smear confirmed CMML (Fig. 1a, III&IV). He died 6 months later from pneumonia in the context of persistent profound anemia (Hb = 6.5 g/dL) and inflammation.

FMF patients have been shown to display high plasma levels of proinflammatory cytokines [9]. Their monocytes are spontaneously activated and secrete high levels of IL-1β [10]. Cytokines implicated in the pathophysiology of FMF such as IL-1β, IL-18 and IL-6 are important for the regulation of immune and inflammatory responses. These cytokines are also involved in the pathophysiology of inflammatory anemia due to erythropoiesis blockade [11, 12]. It is therefore tempting to speculate that the transformation of refractory anemia into CMML, which resulted in an increased number of circulating monocytes in our FMF patient, could be responsible for the persistent inflammatory syndrome. In keeping with this hypothesis, the progressive monocytosis was accompanied by a progressive elevation of CRP levels (Fig. 1b) and persistence of refractory anemia.

We measured the plasma levels of IL-1β, IL-18 and IL-6 in the patient [see Patients and Methods in Additional file 1], 10 months (t = 10) after t = 0, and compared them with cytokine levels from FMF patients carrying unambiguous *MEFV* mutations (n = 8) [see Table S1 for genotype, inflammatory and clinical status of the FMF patients, in Additional file 1] and healthy controls. IL-6, a cytokine known to induce acute phase proteins, was not detected in the controls’ plasma but was present at a concentration of 64 pg/mL in the CMML patient and also easily detectable in 4 other FMF patients (3–177 pg/mL), of whom 3 were under colchicine therapy (Fig. 1c). IL-1β is produced by monocytes and tissue macrophages as a precursor, which is then processed to its active form by the inflammasome, an intracellular multiprotein complex [13]. IL-1β is mainly detected in supernatants of monocyte culture after stimulation with Toll-Like receptor agonists like lipopolysaccharide [10]. Although *ex-vivo* studies demonstrated a major role of IL-1β in the pathogenesis of FMF, serum levels have been reported normal or even decreased in FMF patients [14]. Accordingly, IL-1β was not detected in the patient’s plasma, in controls, and in 6 out of the 8 FMF patients (data not shown). IL-18, which is also secreted by monocytes and regulated by inflammasome, was found at much higher levels in the patient’s plasma (7647 pg/mL) than in other patients (187–4527 pg/mL) or controls (172–246 pg/mL) (Fig. 1d). As these cytokines are known to be involved in chronic inflammation in FMF [9, 10], their presence could readily explain the uncontrolled inflammation seen in the patient.

From a more general viewpoint, our study unveils the interplay between two different disorders involving the same target cells. More specifically, it suggests that in myelodysplasia with inflammatory manifestations [15, 16], mutations in genes causing autoinflammatory syndromes, such as those found in *MEFV*, can be present and thus could be sought [17]. In this context it is interesting to note that an allogenic bone marrow transplantation on a young patient who had congenital dyserythropoietic anemia and FMF was once reported to significantly improve/treat the FMF symptoms [18] due to the hematopoietic involvement of the two disorders.

Our data suggest that among elderly FMF patients, with CMML, a severe inflammatory syndrome may appear and has to be treated. CMML diagnosis should be confirmed by bone marrow aspiration; and cytotoxic chemotherapy and/or a demethylating agent should be considered in order to reduce monocytosis. This is even more important, as monocytosis is a key factor in the pathogenesis of FMF and the subsequent production of proinflammatory cytokines. In these rare cases of FMF or other autoinflammatory diseases combined with CMML, interleukin inhibitors (against IL-1, IL-6 or IL-18) alone or associated with a demethylating agent could represent a valuable therapeutic strategy to decrease a potentially fatal inflammatory syndrome.

## Additional file

Additional file 1:
**Patients and Methods.** **Table S1.** Genotype, inflammatory and clinical status of the FMF patients.

## Abbreviations

FMF: Familial Mediterranean fever
CMML: Chronic myelomonocytic leukemia
CRP: C reactive protein
IL: Interleukin
SAA: Serum amyloid A
