# Supplementary material for: Chronic myelomonocytic leukemia as a cause of fatal uncontrolled inflammation in familial Mediterranean fever
Source: Orphanet J Rare Dis. 2015 Jun 16;10:76. doi: 10.1186/s13023-015-0295-9 (PMC4485869; doi:10.1186/s13023-015-0295-9)
Supplement: Additional file 1: — Patients and Methods. Table S1. Genotype, inflammatory and clinical status of the FMF patients. [file 13023_2015_295_MOESM1_ESM.pdf]

Additional File 1

Patients and Methods

Patients

Nine FMF patients, including the CMML patient, were included in this study, which was performed according to Helsinki convention, and approved by the local Ethics Committee of Necker Hospital. All of them carried unambiguous *MEFV* mutations (Table S1).

Cytokine measurements

Cytokines were measured in the plasma from patients and apparently healthy donors (from the *Etablissement Français du Sang*). Plasma was collected from blood samples after centrifugation and stored at -80 °C at the time of venipuncture. Cytokine levels of all collected plasma samples were measured in the same experiment by ELISA (R&D Systems for IL-1β and IL-6, and MBL for IL-18) according to the manufacturers' instructions.

Statistical analysis

Comparisons were performed using the unpaired Student's t-test. Differences were considered statistically significant at $p<0.05$.

Table S1. Genotype, inflammatory and clinical status of the FMF patients.

**Case No**	***MEFV* mutations**	**Age (years)**	**CRP* (mg/L)**	**SAA* (mg/L)**	**Clinical manifestation**	**Treatment**
1	M694V/M694V	40	2.2	<5	No crisis	Colchicine
2	M694V/M694V	40	88	396	No crisis	No treatment
3	M694V/M694V	57	14.8	9.1	No crisis	Colchicine
4	M694V/M694V	32	20	35	In crisis	Colchicine
5	M694V/M694V	36	9.9	11.5	No crisis	Colchicine
6	M694V/M694V	41	8.3	<5	No crisis	Colchicine
7	M694V/M694I	21	0.9	<5	No crisis	Colchicine
8	M694V/M694V	26	3.0	<5	No crisis	Colchicine
9[§]	M694V/M694V	84	115	226	In crisis	Colchicine

*Normal values, C-reactive protein (CRP) < 5mg/L; serum amyloid A (SAA) < 6 mg/L
§Clinical and inflammatory status of our patient at the time of CMML diagnosis
